# Vibrational spectroscopy to study ancient Roman funerary practices at the “Hypogeum of the Garlands” (Italy)

**DOI:** 10.1038/s41598-022-07689-0

**Published:** 2022-03-08

**Authors:** G. Festa, M. Rubini, P. Zaio, A. Gozzi, N. Libianchi, S. F. Parker, G. Romanelli, L. A. E. Batista de Carvalho, M. P. M. Marques

**Affiliations:** 1 CREF – Museo Storico della Fisica e Centro Studi e Ricerche Enrico Fermi, Rome, Italy; 2 Anthropological Service S.A.B.A.P.-LAZ., Ministry of Culture, Tivoli, Italy; 3 Department of Archaeology, Foggia University, Foggia, Italy; 4 ISIS Neutron and Muon Facility, STFC Rutherford Appleton Laboratory, Chilton, Didcot, OX11 0QX UK; 5 “Molecular Physical Chemistry” R&D Unit, Department of Chemistry, University of Coimbra, 3004-535 Coimbra, Portugal; 6 Department of Life Sciences, University of Coimbra, 3000-456 Coimbra, Portugal

**Keywords:** Applied physics, Characterization and analytical techniques, Infrared spectroscopy

## Abstract

The “Hypogeum of the Garlands” is a sepulchral site, recently found in *Grottaferrata (Lazio*, *Italy*), dating back to the first-second century AD. Two sarcophagi were discovered inside, hosting the human remains of *Aebutia Quarta*, a rich Roman woman, and her son *Carvilius Gemellus*. While the body of *Carvilius* is exceptionally well-preserved, following its embalming and perfect sealing of the sarcophagus, in the case of *Aebutia* only the bones were preserved because of the sarcophagus’s seal breaking down, although she was covered with perfectly preserved flower garlands. Embalming of the body was a rare ritual in the Imperial Roman times when corpses were more often cremated. The remains of *Aebutia* showed possible traces of heating. Burned bones from a third individual were discovered on the chamber’s floor and preliminary anthropological survey showed that this individual was a male of 40–50 years old. Here, a combination of spectroscopic techniques, including non-destructive inelastic neutron scattering and Raman spectroscopy, and minimally destructive Fourier transform infrared spectroscopy, were applied to the analysis of these bone samples to give information about ancient Roman funerary practices. The temperature and burning conditions were thus determined, showing that *Aebutia Quarta* was exposed to mild temperatures (200 °C) only in the upper part of the body, while the third individual was likely cremated as its bones were exposed to temperatures up to 900 °C in quasi-anaerobic conditions.

## Introduction

**Figure 1:**
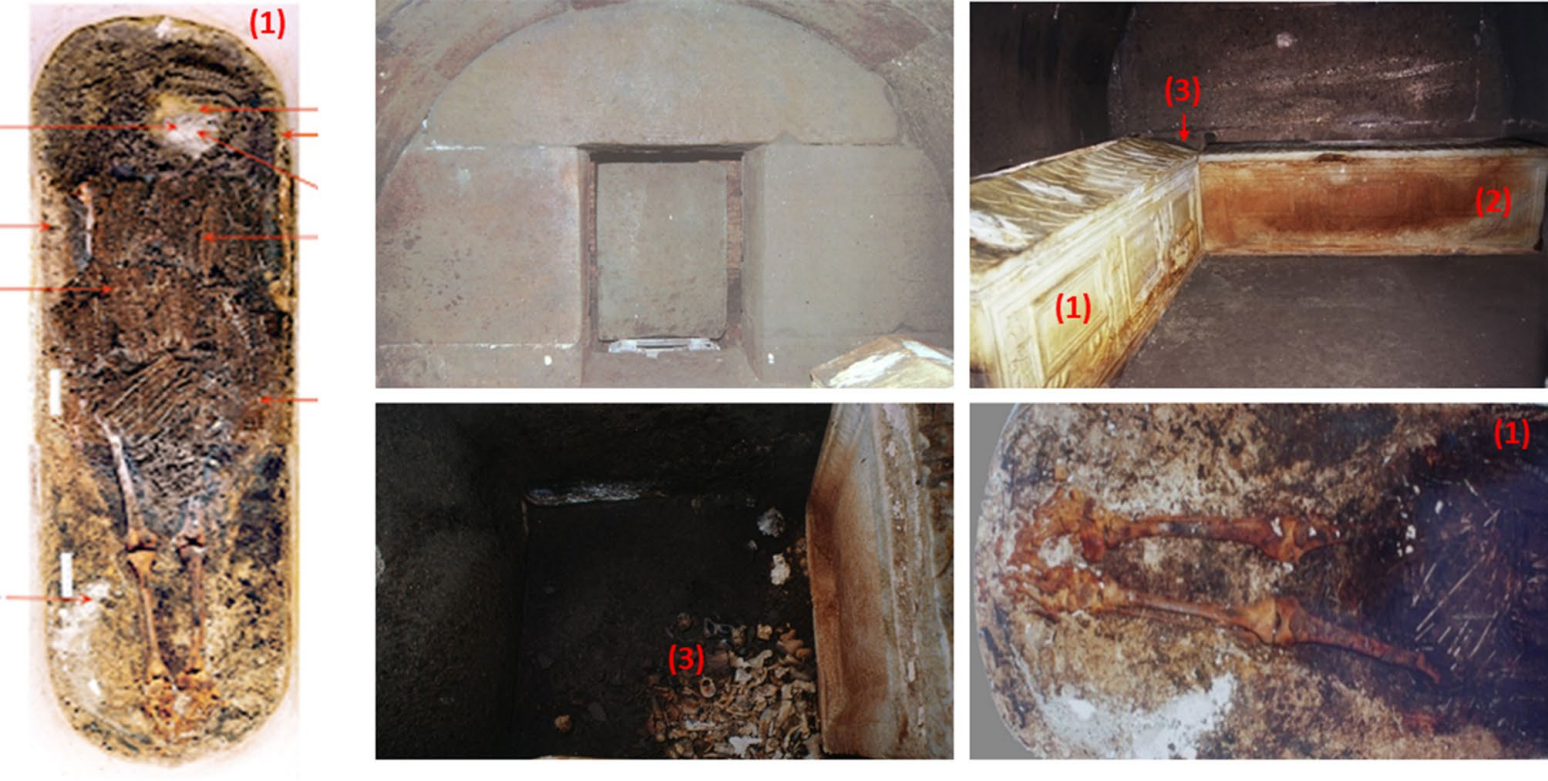
The “Hypogeum of the Garlands”, the sarcophagi of Aebutia Quarta (1) and Carvilius Gemellus (2) and Aebutia skeletal remains; burned bones belonging to A1 individual (3) found on the stone pavement in the corner between the two sarcophagi^1^.

The “*Hypogeum of the Garlands*” is an ancient Roman sepulchral chamber from the Roman Period that has been dated to the first-second century AD^1–4^. It was discovered in 2000 in *Grottaferrata* (Rome, Italy) and represents an extraordinary example of a burial chamber still sealed by the original stone (Fig. 1). Two sarcophagi were found within the hypogeum, of which one contained the exceptionally well-preserved body of about 18 years old *Carvilius Gemellus*, as evidenced by the inscriptions^1^. The second sarcophagus, whose seal was partially broken, contained the skeletal remains of *Aebutia Quarta* (only bones were preserved), mother of *Carvilius*. The age at death has been estimated to be between 32 and 43 years old^1^—through inscriptions and osteological information, additional details may be found in the Material and Methods section. The preservation of the human remains was a consequence the embalming of the bodies, a rare ritual in Roman times when corpses were more often cremated^5^. The onomastic formulae and the funerary architecture show that it belonged to a rich and powerful family from the imperial Roman period. In both cases, the sarcophagi contained still intact vegetal garlands and funerary dresses^6^, as well as a large gold ring and a wig with gold filaments in *Aebutia*^7^. The presence on the skeletal remains of *Aebutia Quarta* of deformations in the diaphysis regions and chromatic variations evident in the upper region of the skeleton have led to the hypothesis by the archaeologists of an atypical high-temperature exposure process, thus boosting speculation about the causes of her death^1,8^. The *Aebutia*’s remains were examined, restored, consolidated and subjected to biocidal treatment after the discovery. They were then stored in an anaerobic environment in sealed plexiglass cases in a controlled atmosphere^8^.

**Figure 2:**
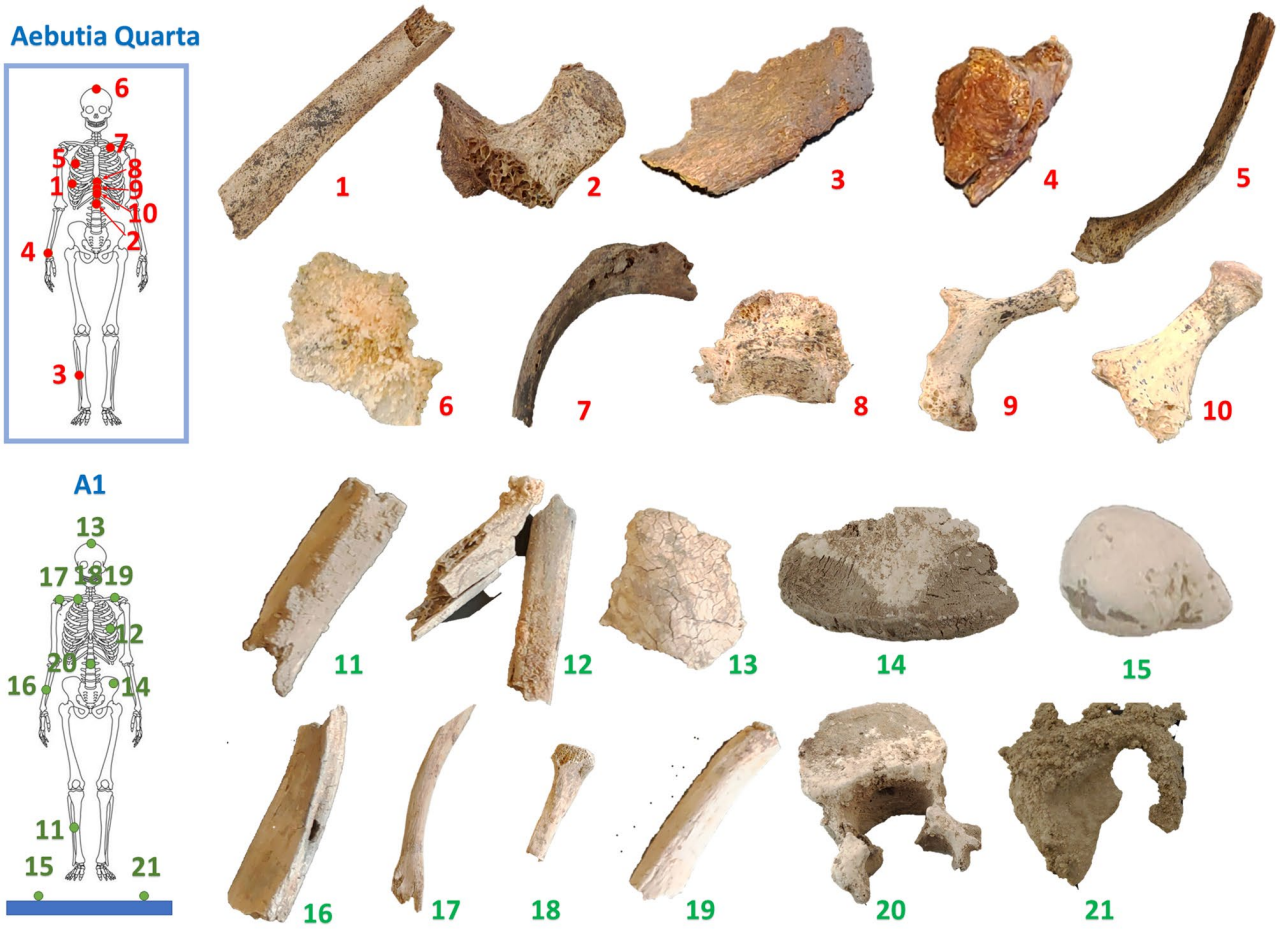
Skeletal remains from the “Hypogeum of the Garlands” studied here. Samples are from Aebutia Quarta and one incinerated individual: AEBUTIA—1. Rib1; 2. Vertebra1; 3. Tibia; 4. Carpal (trapezium); 5. Rib2; 6. Cranium1; 7. Rib3; 8. Vertebra2; 9. Vertebra3; 10. Vertebra4. Individual A1–11. Fibula; 12. Rib4; 13. Craniuml2; 14. Pelvis with white concretions in the upper region; 15. Rock1 fragment; 16. Radius; 17. Clavicle1; 18. Clavicle2; 19. Clavicle3; 20. Vertebra5 with white concretions in the lower region; 21. Rock2 fragment. Details are reported in the “Methods and materials” section.

Some burned bones belonging to a third individual (hereafter referred as A1) were found on the stone pavement, simply stacked in the corner between the two sarcophagi, and no evidence of the use of a container was found. Archaeologists have speculated that they may have been contained in urns made of perishable material^1^. The preliminary anthropological survey (see Material and Methods section) showed that this individual was 40–50 years old and male. Several puzzling questions still surround the story behind *Aebutia Quarta*, including whether she died because of exposure to high temperature, and if this was related to the death of the third individual found between the two sarcophagi. Moreover, the funerary ritual of *Carvilius* and *Aebutia*, involving embalming rather than cremation, seems to differ from the one used in the case of the third individual, possibly suggesting their belonging to a different cult. In order to address these unsolved questions, we investigated the physical–chemical properties of the bone samples found in this hypogeum. Bone is a composite biomaterial comprising packed collagen fibers (mostly type I) woven into an inorganic matrix of crystalline hydroxyapatite (HAp, Ca_10_(PO_4_)_6_(OH)_2_), the hydroxyls being partly substituted by carbonate (ca. 7 wt%)^9–11^). A combination of spectroscopic techniques, including non-invasive inelastic neutron scattering (INS) and Raman spectroscopy, and minimally destructive Fourier transform infrared spectroscopy (FTIR-ATR), have previously been successfully used for the determination of the temperatures and burning conditions of human bones^12–14^. These are complementary vibrational spectroscopy techniques which deliver unique information on the samples under analysis in a virtually completely non-invasive and non-destructive way, enabling chemical and structural changes in bone to be determined with very high accuracy and sensitivity. In this study, we analyze spectroscopic fingerprints to have access to the complete vibrational profile of the bones discovered in *Grottaferrata*. This provides new scientific evidence for the interpretation of ancient Roman funerary practices throughout the “*Hypogeum of the Garlands*” archaeological site. The investigated samples are depicted in Fig. 2.

## Results

**Figure 3:**
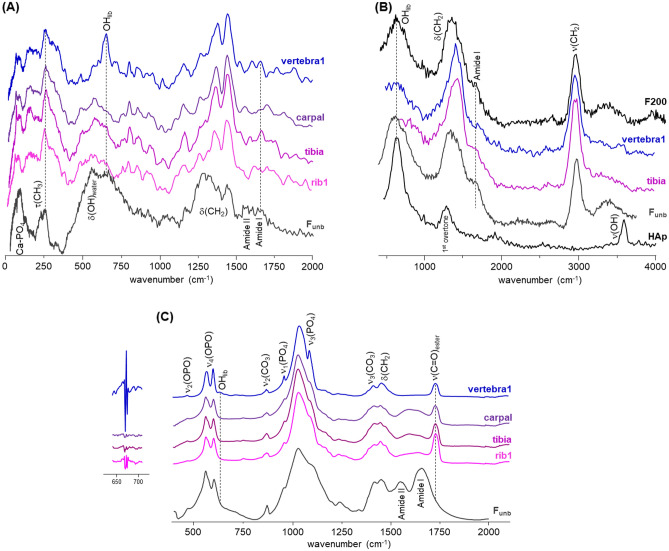
Vibrational spectra of the human skeletal remains from the Aebutia’s sarcophagus (rib1, vertebra1, tibia and carpal): (**A**) INS, measured at TOSCA (0–2000 cm^−1^); (**B**) INS, measured in MAPS (0–4000 cm^−1^, with 5240 cm^−1^ incident energy); (**C**) FTIR-ATR (400–2100 cm^−1^). The second derivative of the data (multiplied by 100), for the 550 to 750 cm^−1^ range, is included in (**C**). The INS and FTIR-ATR spectra of modern human bone (femur), unburned (F_unb_) and burned at 200 °C under controlled aerobic conditions (F200), as well as the INS spectrum of HAp (MAPS, with 5240 cm^−1^ incident energy), are also shown^14–16^.

Figure 3 comprises the INS and FTIR-ATR spectra measured for the skeletal remains found in the *Aebutia’s* sarcophagus (rib1, vertebra1, tibia and carpal, Fig. 2). They are compared with published data previously measured for modern human bones^14–17^, both unburned and burned under controlled conditions (either aerobic or anaerobic), to assist interpretation of the data obtained for the archaeological samples. The INS profiles of rib1, tibia and carpal do not show the characteristic bands of hydroxyapatite (HAp)—OH librational mode at *ca.* 660 cm^−1^ and OH stretching at *ca.* 3600 cm^−1^—while they clearly evidence the presence of organic constituents: lipids—CH_2_ deformations at *ca.* 1370–1450 cm^−1^ and CH_2_ stretching modes at *ca.* 2980 cm^−1^—and traces of collagen—Amide I and II bands at 1650 and 1550 cm^−1^ and the methyl torsion at *ca.* 250 cm^−1^. Previous work has shown that the lipid and protein components of bone disappear on heating to about 400 °C (under oxidizing conditions)^14–16^. From this evidence, one can conclude that rib1, tibia and carpal appear not to have been burned. For the vertebra1 specimen the librational band from Hap’s hydroxyl at 660 cm^−1^ is detected by INS (Fig. 3A) and as a very low intensity signal by FTIR-ATR (Fig. 3C), which indicates that this specimen was subject to some degree of heating (at around 200–300 °C by comparison with reference modern samples, Fig. 3B). Furthermore, these bone fragments (samples 1 to 4) display a very well-defined infrared peak at 1725–1730 cm^−1^ (Fig. 3C), characteristic of fatty acid esters, which may be due to contamination from oils or waxes used in embalming funerary practices. From these results, one can conclude that only the central area of *Aebutia*’s body (vertebrae) was exposed to a mild heat source at ca. 200 °C. Moreover, the presence of intact and well-preserved garlands in the same sarcophagus makes unrealistic any scenario whereby a burning process started within the sarcophagus itself.

**Figure 4:**
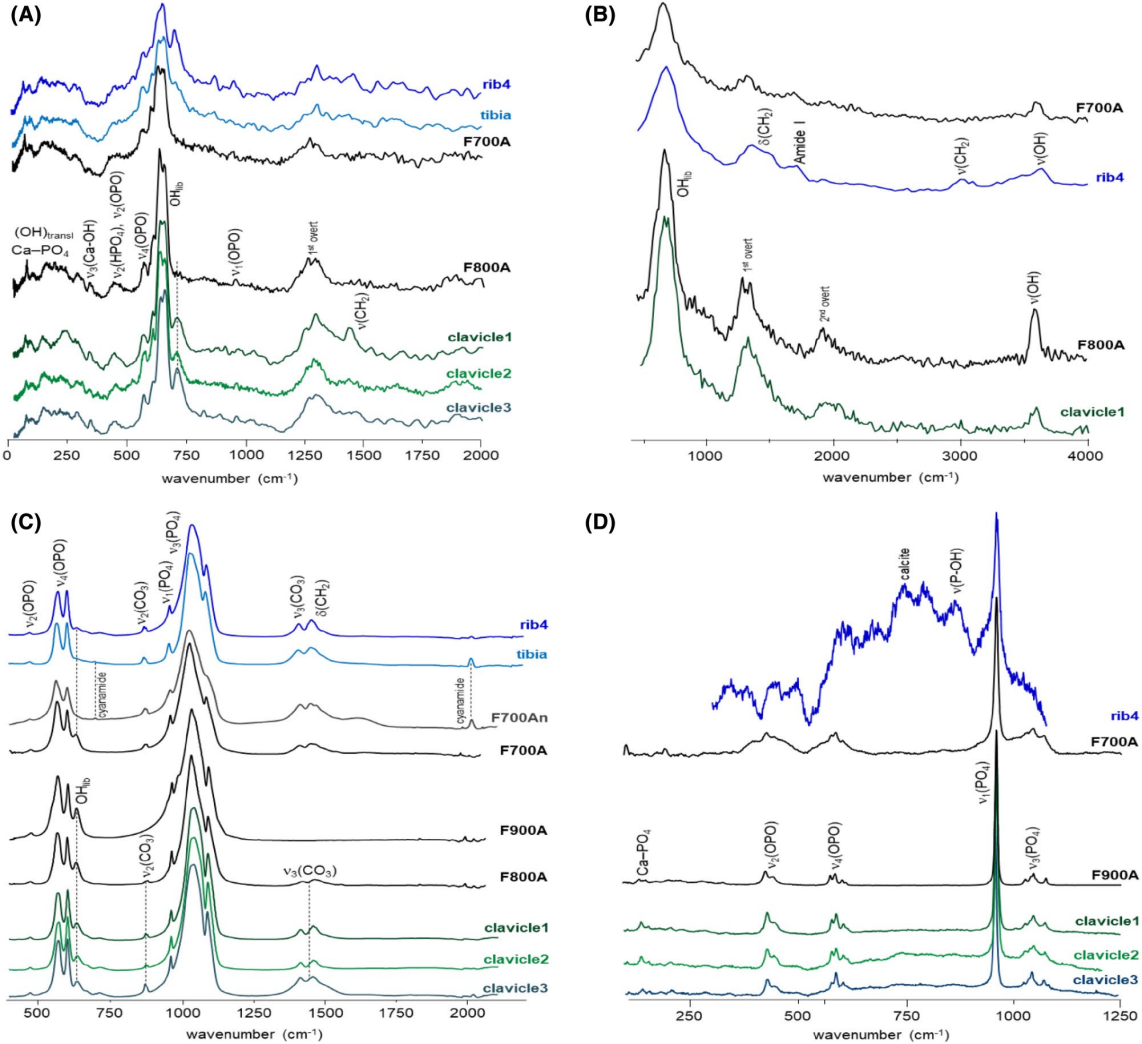
Vibrational spectra of human skeletal remains from individual A1 (fibula, rib4, cranium2, pelvis, radius, clavicle1, 2 and 3): (**A**) INS, measured at TOSCA (0–2000 cm^−1^); (**B**) INS, measured at MAPS (0–4000 cm^−1^, with 5240 cm^−1^ incident energy); (**C**) FTIR-ATR (400–2100 cm^−1^); (**D**) Raman (100–1250 cm^−1^). The INS and FTIR-ATR spectra of modern human bone (femur) burned at 700 °C (F700), 800 °C (F800) and 900 °C (F900), under controlled aerobic (A) or anaerobic (An) conditions, are also shown^14–16^.

As to the remains collected from A1, distinct heating conditions were unveiled, in particular, by the INS data (Fig. 4A,B). While the fibula and rib4 appear to have been subjected to temperatures of *ca.* 700 °C, specimens from the radius, cranium2 and clavicles1, 2 and 3 were burned at higher temperatures. This is corroborated by the FTIR-ATR and Raman spectra, the infrared OH libration being detected for rib4, clavicles 1, 2 and 3, radius and cranium2 (at *ca.* 660 cm^−1^) but not for the fibula (Fig. 4C), while the Raman spectra of the clavicle1, radius and cranium2 comprise the typical narrow and intense ν_1_(PO_4_) phosphate signal from hydroxyapatite (typical of high burning temperatures) as opposed to the spectrum from the rib (Fig. 4D). These vibrational profiles are compatible with a heating temperature of about 800–900 °C. Additionally, there is a noticeable variation in the relative intensity of the Raman bands at 480 and 490 cm^−1^ from the triply degenerate phosphate mode (ν_4_(OPO), Fig. 4D), evidencing the presence of distinct hydroxyapatite polymorphic phases, in different amounts in each of these samples. This indicates that the bone fragments from the clavicle, radius and cranium2 were not subject to exactly the same burning temperature (although always ≥ 800 °C). These skeletal remains also display a band at 725 cm^−1^ (Fig. 4A) which is ascribed to contamination with calcite. Since calcite was only detected for these skeletal remains found directly on the soil (and not for those found inside the sarcophagus), it may be suggested that this contamination is due to water percolation or to a bone treatment, such as the use of lime in funerary practices (see, e.g., Ref.^18^).

Spectroscopic results reveal that the heating processes have occurred in an environment with a reduced oxygen availability (quasi-anaerobic conditions), as demonstrated by the presence of carbonate and cyanamide in the tibia sample as clearly evidenced by FTIR-ATR (Fig. 4C). The inclusion of cyanamide anion (NCN^2−^) within the bone´s framework, identified through its infrared peaks at 702 and 2017 cm^−1^ (respectively from NCN deformation and stretching modes), was previously only reported in bone heated at 650 °C and above under anaerobic conditions^14,19^. These observations lead us to conclude that the oxygen availability during heating differed for these archaeological skeletal remains: the fibula was burned in a more confined and anaerobic environment, leading to the absence of hydroxyapatite´s OH librational band and to the appearance of cyanamide. In addition, rib4 displays a quite intense Raman band at *ca.* 725 cm^−1^ (Fig. 4D), which may be ascribed to calcite (CaCO_3_)^13,20,21^.

**Figure 5:**
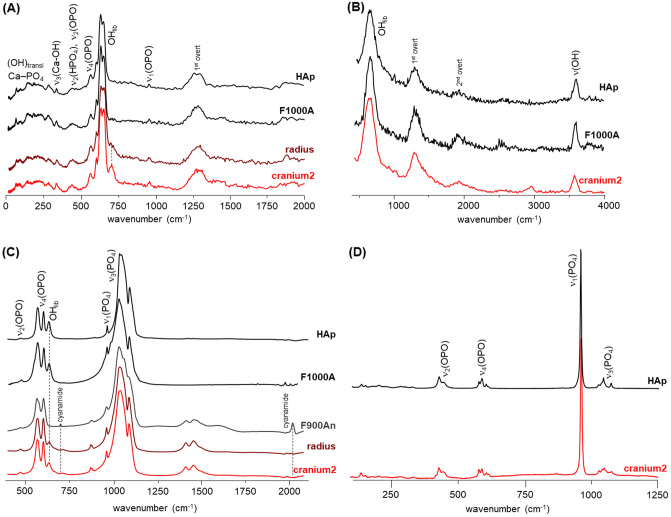
Vibrational spectra of human skeletal remains from individual A1 (radius and cranium2): (**A**) INS, measured at TOSCA (0–2000 cm^−1^); (**B**) INS, measured at MAPS (0–4000 cm^−1^, with 5240 cm^−1^ incident energy); (**C**) FTIR-ATR (400–2100 cm^−1^); (**D**) Raman (100–1250 cm^−1^). The INS and FTIR-ATR spectra of modern human bone (femur) burned at 900 °C (F900) and 1000 °C (F1000), under controlled aerobic (A) or anaerobic (An) conditions, as well as the INS, FTIR-ATR and Raman profiles of reference calcium hydroxyapatite (SRM 2910b, HAp), are also shown^14–16^.

**Figure 6:**
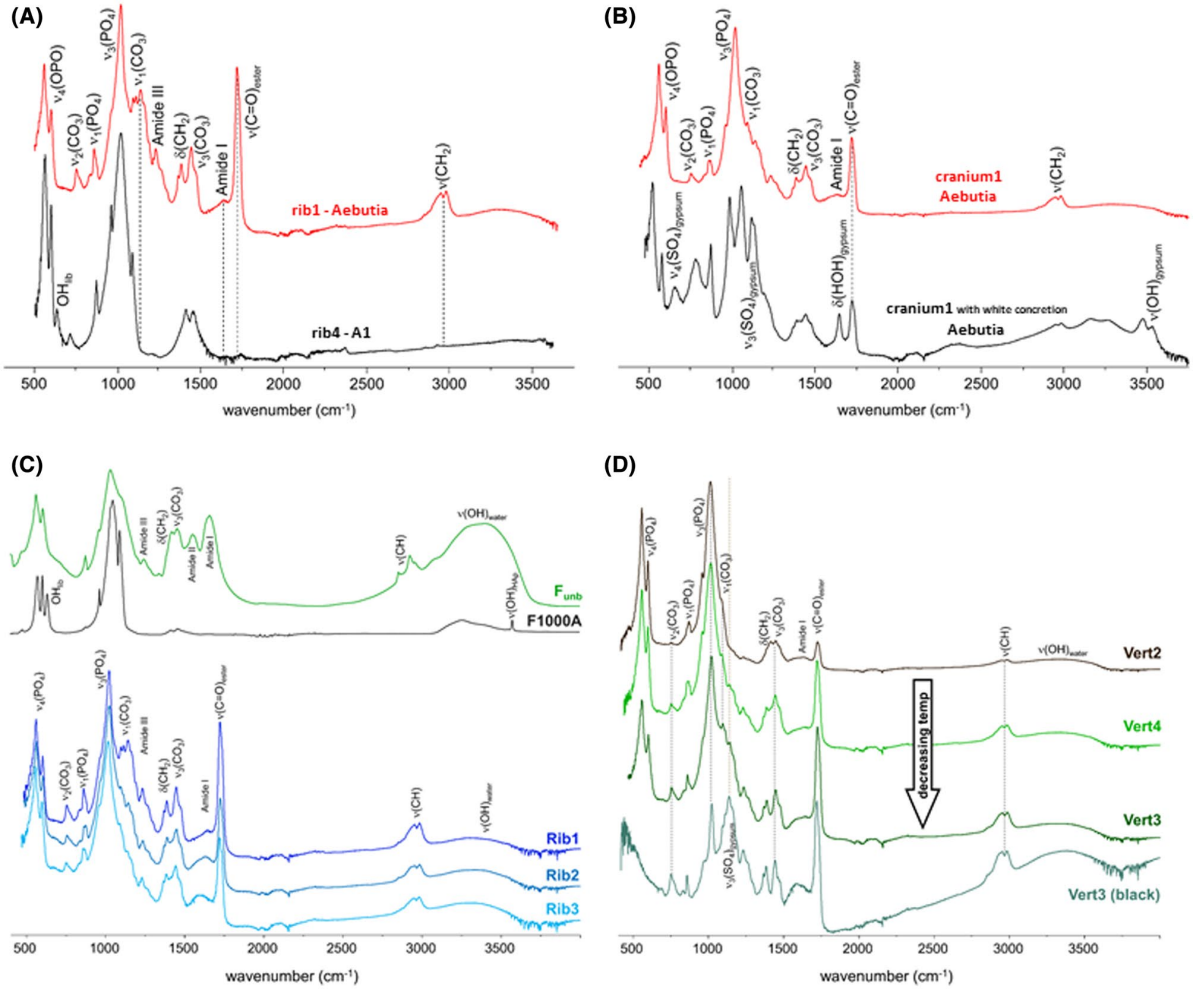
FTIR-ATR spectra (400–3750 cm^−1^) of human skeletal remains: (**A**) rib1 fragment from the Aebutia’s sarcophagus and A1; (**B**) cranium1 specimens from the Aebutia’s sarcophagus, with and without white concretions; (**C**) rib1, 2, 3 fragments from Aebutia’s sarcophagus and comparison with modern human bone (femur) burned at 1000 °C (F1000) and unburned; (**D**) comparison of FTIR-ATR spectra of vertebrae2, 3, 4 from Aebutia’s sarcophagus.

**Figure 7:**
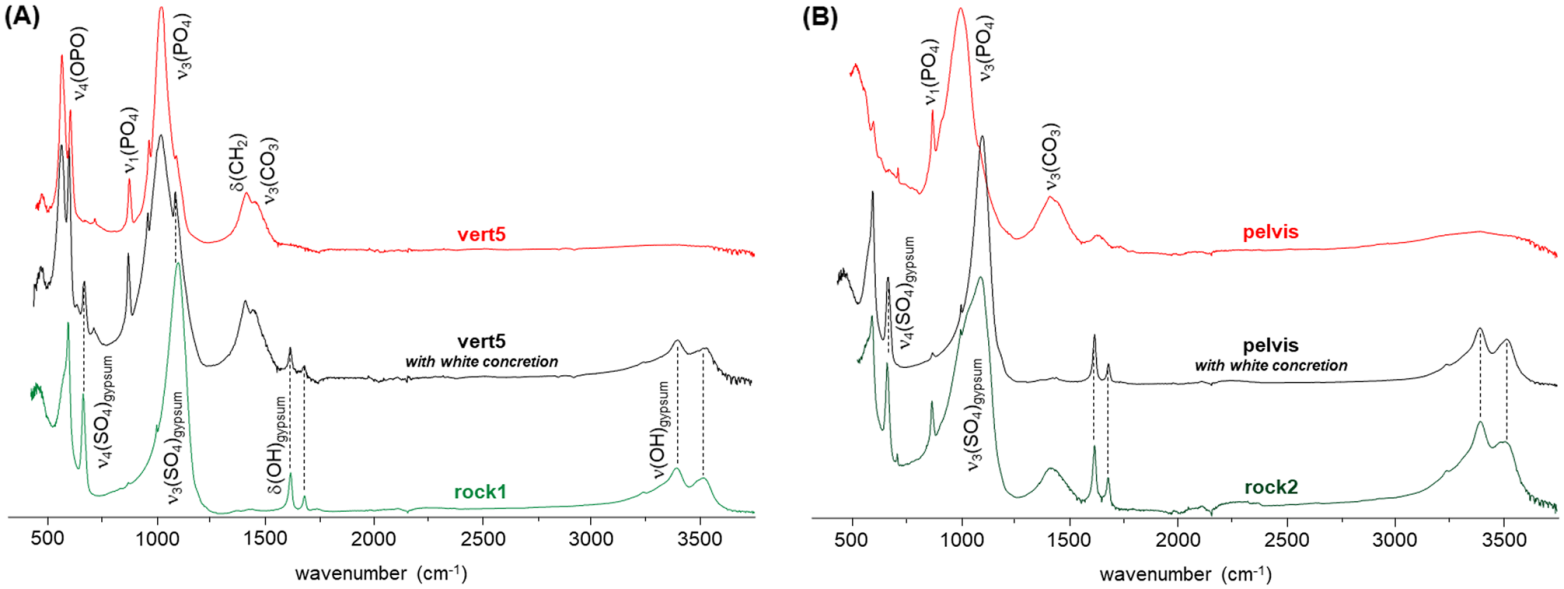
FTIR-ATR spectra (400–3750 cm^−1^) of human skeletal remains from individual A1 (with and without white concretions): (**A**) vertebrae5 specimens; (**B**) pelvis specimens. The spectra of fragments of white rock1 fragment1 and of the rock2 fragment beneath the bones are also shown.

Regarding samples from the radius and cranium2 from individual A1, they appear to have been exposed to the highest temperatures, above 900 °C (Fig. 5). The corresponding INS profiles show a well-defined and intense OH librational band, displaying a close similarity to those from modern human femur burned at 1000 °C and from the HAp reference material (Fig. 5A,B). This is corroborated by the FTIR-ATR and Raman spectra obtained for these ancient specimens (Fig. 5C,D). The fact that no cyanamide is detected for either of these skeletal remains is indicative of an oxidative (aerobic) burning environment^14^. Further FTIR-ATR measurements were collected for bones belonging to *Aebutia* and the individual A1, to reinforce and generalize what has been found for the first set of skeletal fragments. The results are shown in Figs. 6 and 7. Figure 6A reports the infrared spectra of human rib remains found both inside the *Aebutia’s* sarcophagus and on the floor (from individual A1).

Figure 6C and D shows the FTIR-ATR spectra of rib fragments and vertebrae from the *Aebutia’s* sarcophagus, in comparison with modern human bone (femur) unburned and burned at 1000 °C. The signals from the ancient ribs show an intense band at *ca.* 1720 cm^−1^, evidencing the presence of esters from fatty acids due to contamination from oils or waxes used for embalming the corpses. The characteristic δ(CH_2_) signals from protein and lipids, as well as from carbonates, are also visible, together with low intensity bands from amide I (ν(C=O)) and II (ν(C=O) + δ(C-NH)) from collagen. Neither the OH librational nor stretching bands are detected, suggesting a low heating temperature. No cyanamide formation is observed, which points to an aerobic environment. In the *Aebutia’s* vertebrae, a decrease in the intensity of the band at *ca.* 1720 cm^−1^ (esters from fatty acids) is observed, evidencing a difference in the degree of contamination from oils/waxes along the skeleton. Also, the amount of carbonate and organic components increases, as a consequence of decreasing heating temperatures. These lower temperatures also explain the higher amount of oils in these samples (ν(C=O) at 1720 cm^−1^). The signal at *ca.* 1140 cm^-1^, detected only for vertebra3 in a blackened region of the bone fragment (Fig. 6D), is tentatively ascribed to the ν_3_(SO_4_) mode from gypsum (CaSO_4_·2H_2_O), quite intense in the FTIR-ATR spectrum. This has been previously observed (using Raman spectroscopy) for archaeological bones^17^ and may be indicative of contamination from the limestone soil. Water percolation from the environment into the *Aebutia’s* sarcophagus, which is made of marble (mostly CaCO_3_), could have led to the formation of gypsum and subsequent contamination of some of the skeletal remains found inside this sarcophagus in the upper part (in the form of white concretions)—this was observed only for one cranium1 specimen, which also showed the presence of oils/waxes along with the δ(HOH) signal from gypsum (at *ca.* 1718 cm^−1^) (Fig. 6B).

In contrast, the samples from A1 often showed the presence of gypsum (CaSO_4_.2H_2_O), mainly those with white concretions attached to the specimen, as clearly seen when comparing two samples from the same type of bone (Fig. 7). The characteristic signals from CaSO_4_·2H_2_O are clearly detected at 596, 663, 1620, 1680, 3390 and 3520 cm^−1^, assigned to ν_4_(SO_4_), ν_3_(SO_4_), δ(HOH) and ν(OH_2_), respectively^22^. This contamination can be due to the soil composition, since rock samples retrieved from the site (e.g. sample 15) and fragments found beneath the bones showed an infrared profile compatible with a major composition being gypsum (CaSO_4_·2H_2_O, Fig. 7).

## Discussion

A key point in the study of ancient civilizations is the way they dealt with human remains after death. Between the fourth century BC and the first century AD, the cremation rite was prevalent in Ancient Roman civilization and inhumation was rare^23^. By comparing the results obtained here for the archaeological samples with published data previously gathered for modern human bones burned under controlled conditions (regarding temperature and oxygen availability)^14–16^, it is possible to estimate the temperatures to which the investigated archaeological samples were subject to. Table 1 summarizes the information gathered from the analysis of the experimental results from the previous section: (i) samples 2, 5, 7, 8, 9 and 10 found inside *Aebutia’s* sarcophagus, located in the central area of the skeleton, were subject to very mild heating (*ca.* 200 °C at maximum), while *Aebutia’s* rib1, tibia, carpal and cranium1 were unburned; (ii) bones from A1 appear as burned—at ca. 700 °C for fibula, rib4 and vertebra5, at ca. 800 °C for pelvis, clavicle1, 2 and 3, and at temperatures higher than 900 °C for cranium2 and radius.

**Table 1:** Archaeological skeletal remains analyzed in the present study and overall heating conditions and obtained results.

Site	N	Sample	Dimensions (cm^3^)	Heating temperature		Heating conditions (oxygen availability)	Observations
AEBUTIA	1	Rib1	7.0 × 1.5 × 1.0	Unburned	–	–	Fatty acid esters (from oils or waxes) Small white concretions on the cranium—gypsum
	2	Vertebra1	4.0 × 4.0 × 1.5	Burned	ca. 200 °C	Aerobic	
	3	Tibia	6.0 × 2.5 × 1.0	Unburned	–	–	
	4	Carpal	3.0 × 2.0 × 2.0	Unburned	–	–	
	5	Rib2	7.0 × 1.0 × 0.8	Unburned	–	–	
	6	Cranium1	4.5 × 3.0 × 0.5	Unburned	–	–	
	7	Rib3	5.5 × 1.0 × 0.5	Burned	ca. 200 °C	Aerobic	
	8	Vertebra2	3.5 × 2.0 × 1.0	Burned	ca. 200 °C	Aerobic	
	9	Vertebra3	4.5 × 1.5 × 0.8	Burned	ca. 200 °C	Aerobic	
	10	Vertebra4	4.0 × 2.5 × 1.0	Burned	ca. 200 °C	Aerobic	
A1	11	Fibula	5.0 × 1.5 × 1.0	Burned	ca. 700 °C	Anaerobic	Highly confined and reductive environment. Presence of cyanamide Presence of calcite (CaCO_3_) and gypsum (CaSO_4_·2(H_2_O)) also in the rock fragment 1 and 2 Confined and reductive environment
	12	Rib4	8.0 × 1.0 × 0.8	Burned	ca. 700 °C	Quasi-anaerobic	
	13	Cranium2	3.5 × 4.0 × 1.0	Burned	> 900 °C	Quasi-anaerobic	
	14	Pelvis	13.0 × 5.0 × 4.0	Burned	ca. 800 °C	Anaerobic	
	15	Rock1	2.0 × 2.0 × 1.0	–	–	–	
	16	Radius	5.0 × 1.5 × 2.0	Burned	> 900 °C	Quasi-anaerobic	
	17	Clavicle1	6.0 × 2.0 × 1.5	Burned	ca. 800 °C	Aerobic	
	18	Clavicle2	4.0 × 2.0 × 1.5	Burned	ca. 800 °C	Aerobic	
	19	Clavicle3	5.0 × 1.0 × 2.0	Burned	ca. 800 °C	Aerobic	
	20	Vertebra5	6.0 × 3.0 × 4.0	Burned	ca. 700 °C	Anaerobic	
	21	Rock2	10.0 × 6.0 × 4.0	–	–	–	

Overall, the skeletal remains of individual A1—laid on the floor—were subject to much higher temperatures, as expected, as a consequence of cremation. Nevertheless, it should be pointed out that the modern skeletal remains taken as references in these types of studies are burned as dry bones (defleshed). Hence, it may be argued that the results thus obtained do not take into account the protective effect of the soft tissues towards the impact of temperature on the bone. Comparison between defleshed (dry) burned bones and archaeological skeletal remains subject to burning has been found to be a valid approach to model archaeological findings^15^ and comprehensively assess alterations in bone´s chemical composition and crystallinity. It should be noted that the INS technique probes the bulk of the bone, owing to the high-penetration power of neutrons, thus providing a chemical-physical information on the entire bone rather than related to its surface only. Therefore, while our results can be used to estimate the maximum temperature of exposure but not its duration, in the case of the individual from the second burial we can assume that the high-temperature exposure was long enough to provoke a modification of the entire bone, compatible with a cremation process. Finally, it is worth noting that hypogea and sealed burial chambers were meant to accommodate human remains subjected to different funeral rituals (cremation, burial, embalming, etc.) following individual desire and religious practices. In some cases, especially for rich Roman families, the deceased’s body was displayed in the family’s house for up to 7 days^5^. In this scenario, an embalming process, such as the one affecting the human remains of *Aebutia Quarta* and *Carvilius Gemellus*, could be related to the attempt to better preserve the body of these members of an important family during such display.

## Conclusions

In this study, we have investigated the possible burning conditions, including the maximum temperature, to which skeletal remains from the “*Hypogeum of the Garlands*” were exposed, using a combination of vibrational spectroscopic techniques—inelastic neutron scattering (INS), Fourier transform infrared (FTIR-ATR) and Raman spectroscopies for the determination of the temperatures and the burning conditions of the bones. Regarding the remains of *Aebutia*, the results exclude a burning process, despite the presence of deformation and blackening of some skeletal areas*.* Our results confirm that *Aebutia* underwent an embalming process by applying oils or waxes, which were identified through the presence of spectral biomarkers from fatty acid esters. Furthermore, the central area of the body shows signs of exposure to a low temperature and localized heat source (< 200 °C). As to the remains collected from A1, distinct heating conditions were unveiled according to the INS and FTIR-ATR data, showing burning temperatures that span from *ca.* 700 °C to above 900 °C. The oxygen availability during the heating events differed for these distinct skeletal remains yet suggesting a prevalently anaerobic burning compatible with cremation of the body. In addition, no traces of oils/waxes are detected for this individual, with respect to *Aebutia Quarta* and *Carvilius Gemellus* that did not entail embalming of the corpse.

In conclusion, the vibrational spectral data gathered for these samples (both optical and neutron-based) allow us to retrieve important and unique information regarding burial habits and ancient Roman funerary practices. Furthermore, this methodological investigation can provide objective data that is complementary to the use of the classic chromatic scale for determining the maximum temperature reached.

## Materials and methods

### Materials

The archaeological human bone samples studied here are listed in Table 1 and shown in Fig. 2. Prior to spectroscopic analysis, gentle mechanical removal of the bones´ outer layer was carried out to avoid contaminants. The bone fragments were selected to be in significant numbers for the two studied individual: *Aebutia Quarta* and A1. *Aebutia’s* bones were discovered in anatomical connection and highlighted possible trace of heating only in the upper region of the skeleton associated with possible thermal deformations; for this reason, the investigations are focused in this area (see Fig. 2). The bones of a second individual, named as A1, were found piled up on the floor and present evident heating traces; in this case the measurements are equally distributed along the skeleton.

For the female individual *Aebutia Quarta*, the removal of the flower garland has highlighted possible traces of heating, associated with possible thermal deformations. These heating-like alterations are localized on the anterior area between the cranium and the proximal half of the femora. The cranium, the vertebrae and the pelvis are involved in a process of fragmentation and micro-fragmentation associated with a slight chromatic variation that change from gray brown to black. The deformations involve only the diaphyses of the long bones (humeri and femora). The biological diagnosis of sex agrees with the epigraphic data and with the presence of a typically female wig with a gold net. The osteological analysis was performed according to the criteria standardized by the scientific community^24–26^. Whenever possible, morphometric variables with a discriminating function have been used for the cranium and pelvis^27–29^. Furthermore, an evaluation related to the development of the *linea aspera* of the femur and a multivariate analysis of metric characters relevant to some skeletal areas such as the humerus, the femur and the tibia were carried out^30–33^. For the biological diagnosis of the age at death, the percentage of migration of the cancellous bone in the proximal epiphysis of the humerus and femur was evaluated^34^ in association with the alterations of the pubic symphysis^35^. The analysis of the sutural obliteration^36^ and the degree of dental wear^37^ have been cautiously taken into consideration. The biological profile (sex and age) of the male individual *Carvilius Gemellus* was confirmed, with respect to the epigraphic data (18 years and three months) at about 18 years old, through non-destructive industrial computer tomography (industrial CT). The tomographic examination, with very high-definition images, allowed the reconstruction of the anatomical areas and therefore the biological identification of the different degrees of skeletal ossification^26,38,39^ and of sexual dimorphism^26,40^. The osteological analysis of A1 was carried out on the identifiable skeletal portions and attributed to the same individual and was performed, as for *Aebutia Quarta* and *Carvilius Gemellus*, according to the standard methods^24,36,41^.

### Spectroscopic measurements

#### INS spectroscopy

The INS measurements were carried out at the ISIS Pulsed Neutron and Muon Source of the STFC Rutherford Appleton Laboratory (United Kingdom), using the time-of-flight high resolution broad range TOSCA^42,43^ and MAPS^44–46^ spectrometers. The intact archaeological bones were wrapped in aluminium foil and fixed onto (4 × 5 cm) flat Al frames with Al tape and (as previously reported^17^). To reduce the impact of the Debye–Waller factor on the observed spectral intensity, the samples were cooled to 5–10 K. Data were recorded in the energy range 0 to 4000 cm^−1^, and converted to the conventional scattering law, S(Q,ν) *vs* energy transfer (in cm^−1^) using the MANTID program (version 5.1)^47^.

#### FTIR-ATR spectroscopy

FTIR-ATR data was measured in attenuated total reflectance (ATR) mode, at the “Molecular Physical-Chemistry” R&D Unit of the University of Coimbra (QFM-UC, Portugal)^48^, using a Bruker Optics Vertex 70 FTIR spectrometer purged by CO_2_-free dry air and a Bruker Platinum ATR single reflection diamond accessory. A liquid nitrogen-cooled wide band mercury cadmium telluride (MCT) detector and a Ge on KBr substrate beamsplitter were used for the mid-IR interval (400–4000 cm^−1^). These data were obtained for very small bone splinters, carefully scraped from each bone sample in the fracture area. 128 scans were summed for each spectrum, at 2 cm^−1^ resolution, applying the 3-term Blackman–Harris apodization function, yielding a wavenumber accuracy better than 1 cm^−1^. The Opus 7.2 software was used to correct the spectra regarding the wavelength dependence of the penetration depth of the electric field in ATR, using a mean refractive index of 1.25.

Additional FTIR measurements were collected in attenuated total reflectance (ATR) mode, at the CREF^49^, using a NICOLET iS5 spectrometer (Thermo Fisher Scientific) by the ConservatIR™ external reflection accessory with the ATR sampling head^50^ on the intact samples in the fracture area. Spectra were sequentially recorded between 4500 cm^–1^ and 400 cm^–1^ with a resolution of 2 cm^–1^. The spectra were recorded at room temperature with 128 scans to reduce the background noise.

#### Raman spectroscopy

Raman spectra were obtained, for the intact archaeological bones, at the “Molecular Physical-Chemistry” R&D Unit of the University of Coimbra (QFM-UC, Portugal)^48^, in a WITec confocal Raman microscope system alpha300R, coupled to an ultra-high throughput spectrometer 300 VIS grating (f/4 300 mm focal length, 600 lines *per* millimetre blazed for 500 nm). The detection system was a 1650 × 200 pixels thermoelectrically cooled (– 55 °C at room temperature) charge-coupled device camera, front-illuminated with NIR/VIS antireflection coating, with a spectral resolution < 0.8 cm^–1^/pixel. The excitation radiation used was a WITec 785 nm diode laser, *ca.* 20 mW at the sample position was applied. A 10 × objective (Zeiss Epiplan, NA 0.23, WD 16.1 mm) was used. 20 accumulations were collected *per* sample, with 30 s exposure time. Bone tissue often displays autofluorescence^51^ and fluorescent aromatic compounds may be formed during burning (mainly under anaerobic conditions)^52,53^. This complicates Raman acquisition as fluorescence often masks the Raman signals, mainly for samples not subject to heat or burned at lower temperatures. The use of a near-infrared 785 nm laser enabled us to overcome this problem, providing good quality Raman data for most of the archaeological samples under study.

### Ethics statement

Handling and investigation of the archaeological samples were conducted within the procedural guidelines of the curating organization, and none of the techniques used in this work are considered to be destructive in nature. Measurements were duly authorized by the Central Institute and by Professor *Mauro Rubini* (Anthropology Service, *Soprintendenza Archeologica, Belle arti e Paesaggio per le Province di Frosinone e Latina SABAP-LAZIO*, MIC—*Ministero Italiano della Cultura*) co-author of the paper and the one who formally authorized the essentially non-destructive analysis of ancient finds in full compliance with the ethical rules approved by the Italian Anthropological Association.
